# Extraction Of Transvenous ICD Leads In An Over-ninety Years Old Patient

**Published:** 2011-10-02

**Authors:** Riccardo Proietti, Leonida Lombardi, Carlo Quaglia, Antonio Sagone

**Affiliations:** Cardiac Electrophysiology Laboratory Luigi Sacco Hospital Milano

**Keywords:** lead extraction, PM extraction

## Abstract

There is a general consensus that once a part of an implanted cardiac device becomes infected, it is usually impossible to cure the infection without completely removing all prosthetic material from the body. Consequently the Heart Rhythm Society (HRS) included the pocket infection or erosion as a class I indication for pacemaker lead exctraction. However, the procedure still carries a high risk of life-threatening complications due to fibrotic attachments between leads, veins, valves or other endocardial structures,  notwithstanding specific tools and techniques that have been developed to assist the lead removal, preventing tissue laceration.

Typically, in clinical practice, device removal is often delayed in favor of initial management with antimicrobial therapy. This approach usually performed in elderly can confer an ominous prognosis. We report a case of successful multiple leads extraction in an over ninety year old patient with cardiac device infection resulting in a severe sepsis while treated with antimicrobial therapy.

## Case report

A ninety-two years old patient, affected by idiopathic dilated cardiomyopathy with ejection fraction 21%, class NYHA IV and LBB at ECG (QRS 145 msec) was implanted with CRT-D (Guidant Contact Renewal with Sprint Fidelis lead) six year earlier. Three years after implantation the device was replaced electively. Two years later, due to lead failure with recurrent inappropriate shocks, a new lead (single coil) was positioned in the right ventricle and the Sprint Fidelis lead was abandoned. A few months after the operation the patient presented with severe and extensive pocket infection, treated by pocket revision with extensive necrotic tissue excision. The new defibrillating lead in the right ventricle was abandoned and a CRT-P device was connected to the lead in the coronary sinus. One month after, the patient was referred to our institution by the family doctor because of recurrent fever and shivering since ten days. He was treated with antibiotics (amoxicillin plus clavulanic acid) without benefit.

**Figure 1:**
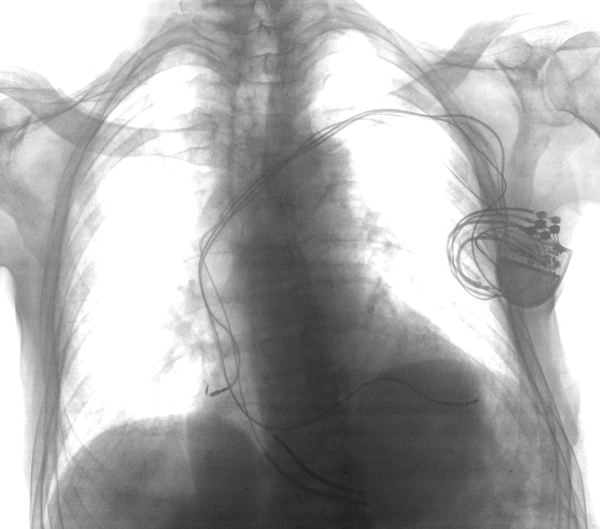
Chest X-ray before the procedure showing two electrocatheters in the right ventricle, one in the coronary sinus and a right auricular catheter.

On admission the patient was febrile (39ºC) hypotensive (BP 90/60 mmHg) and tachycardic (HR 95 bpm). Physical inspection revealed clear lung fields, fast heart rate with 2/6 systolic ejection murmur and mild pretibial edema. The skin over the pace-maker pocket was red and warm. Blood analysis showed significant increase of inflammatory markers  (White blood cells 30000/mm^3^, C Reactive Protein 260 mg/L). A chest X ray showed no infiltrates and enlarged heart shadow (Figure 1). The EKG revealed sinus rhythm at 95 bpm with constant left-ventricular pacing. Hemoculture were drawn before vancomycin therapy was started. At a soft tissue echography of the inflamed area, a fluid collection of 9 x 6 x 40 mm was present. Transthoracic echocardiography showed marked biventricular dilatation, with depressed ejection fraction and moderate mitral regurgitation. The absence of intracardiac vegetation was confirmed by transesophageal echocardiography.

Hemocultures were positive for Staphylococcus aureus (non MRSA) and the patient was treated with multiple antimicrobial agents (vancomycin-teicoplanin-rifampin-oxacillin-gentamicin), because of sepsis and soft tissue infection in a patient with implanted device. However, after more than one week of antimicrobial therapy, remitting fever was still present although the inflammatory markers consistently decreased; echocardiographic follow-up was persistently negative for vegetation. At this time we decided to proceed to lead extraction after obtaining informed consent.

Radioscopy before the procedure, showed two electrocatheters in right ventricle, one in coronary sinus and a right auricular catheter. A cardiac surgery operating room was on stand-by. Under local anesthesia, the pacemaker pocket was opened revealing massive purulent discharge. The extremities of defibrillation coils and sensing and pacing of sprint fidelis electrocatheter were capped together. All leads were been isolated and straightened until the venous entry site. A locking stylets to enable counter-traction is advanced until the top of the electrocatheter (Liberator™, Leechburg, PA, USA) in every single lead while proceeding with the extraction.

The Sprint fidelis electrocatheter and catheter in coronary sinus were removed by manual traction. A non-powered  Evolution™ sheath  (Cook Medical Inc) 9F was used for extraction of  atrial and ventricular sensing and pacing electrocatheter. The Evolution sheath was advanced over the lead until the tip of the electrocatheter in order to mechanically disrupt the fibrosis and create sufficient room to remove the lead.

**Figure 2:**
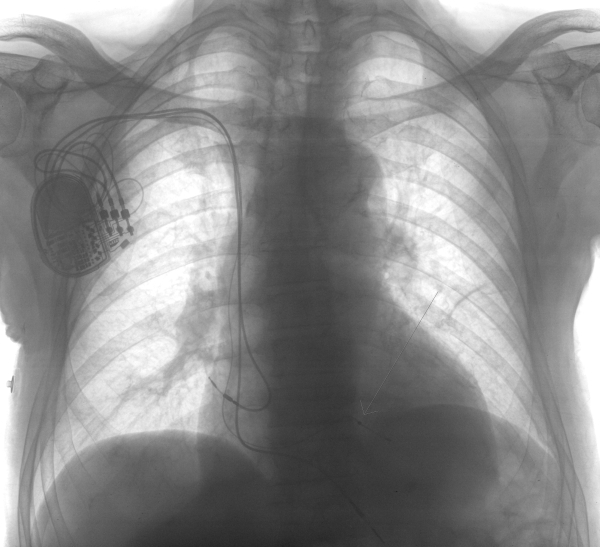
Chest X-ray showing the final re-implant from the right side

Toiletry of cardiac pacemaker pocket involving debridement was carried out and local antibiotic therapy was administered. The patient did well during the entire procedure and general anesthesia was not required. No complications occurred during the procedure. Post-operatively the patient did not experience further episodes of fever and blood chemistry panels improved continuously, enabling successful right-sided implantation of ICD-CRT two weeks later (Figure 2). Eventually, the patient was discharged in good condition.

## Discussion

Device removal is mandatory in case of infection [1] as outlined by recent HRS guidelines [2]. However, in clinical practice it is often delayed in favor of initial management with antimicrobial therapy and pocket revision, as clearly appears in the case described.

We suppose that technical difficulties linked to the age of the patient and presence of multiple lead were the reasons to initially follow a conservative approach, which ultimately did not solve the infection. However, it is worth to remark that while the level of difficulty and complication risk of lead extraction is proportional to the number of leads, [3-5] as experienced by the Lead Extraction Registry, the age of the patient has not been shown to be predictor of complications [5]  - even if older patients are more likely to progress to a calcified fibrosis which creates binding sites from which it is very difficult to free the lead. Moreover, recent evidences show that early device removal is critical in the management of leads infection, since delayed operation is associated with a three-fold increase in 1 year-mortality [6].

In our opinion in order to obtain the resolution of the septicemia, the eradication of the infected focus is even more important in elderly vulnerable patients. A critical aspect was definitely characterized by the tools chosen for the extraction process. In our case, the Evolution System was used as first line extraction tool considering the high chance to find severely calcified binding sites, where laser use would be ineffective [7]. Moreover, the Evolution Mechanical Dilator Sheath has a rotational mechanism with a stainless-steel bladed tip to overcome fibrosis and cut adherences [7].

In conclusion, infected lead extraction has no major age contraindication while it maintains its lifesaving clinical role even in the very aged.
